# Effects of Complementary Feeding With Different Protein-Rich Foods on Infant Growth and Gut Health: Study Protocol

**DOI:** 10.3389/fped.2021.793215

**Published:** 2022-01-13

**Authors:** Minghua Tang, Kinzie L. Matz, Lillian M. Berman, Kathryn N. Davis, Edward L. Melanson, Daniel N. Frank, Audrey E. Hendricks, Nancy F. Krebs

**Affiliations:** ^1^Department of Pediatrics, Section of Nutrition, University of Colorado Anschutz Medical Campus, Aurora, CO, United States; ^2^Division of Endocrinology, Metabolism and Diabetes, University of Colorado Anschutz Medical Campus, Aurora, CO, United States; ^3^Division of Infectious Disease, Department of Medicine, University of Colorado Anschutz Medical Campus, Aurora, CO, United States; ^4^Department of Mathematical and Statistical Sciences, University of Colorado Denver, Denver, CO, United States

**Keywords:** growth, gut microbiota, protein, complementary feeding, infant

## Abstract

**Background:** An urgent need exists for evidence-based dietary guidance early in life, particularly regarding protein intake. However, a significant knowledge gap exists in the effects of protein-rich foods on growth and development during early complementary feeding.

**Methods:** This is a randomized controlled trial of infant growth and gut health (primary outcomes). We directly compare the effects of dietary patterns with common protein-rich foods (meat, dairy, plant) on infant growth trajectories and gut microbiota development (monthly assessments) during early complementary feeding in both breast- and formula-fed infants. Five-month-old infants (up to *n* = 300) are randomized to a meat-, dairy-, plant-based complementary diet or a reference group (standard of care) from 5 to 12 months of age, with a 24-month follow-up assessment. Infants are matched for sex, mode of delivery and mode of feeding using stratified randomization. Growth assessments include length, weight, head circumference and body composition. Gut microbiota assessments include both 16S rRNA profiling and metagenomics sequencing. The primary analyses will evaluate the longitudinal effects of the different diets on both anthropometric measures and gut microbiota. The secondary analysis will evaluate the potential associations between gut microbiota and infant growth.

**Discussion:** Findings are expected to have significant scientific and health implications for identifying beneficial gut microbial changes and dietary patterns and for informing dietary interventions to prevent the risk of overweight and later obesity, and promote optimal health.

**Clinical Trial Registration:** www.ClinicalTrials.gov, identifier: NCT05012930.

## Introduction

Undesired growth patterns during infancy, namely rapid weight gain or excessive weight gain relative to length, are strongly associated with childhood obesity (1, 2). Overweight and obese children have an increased risk of becoming overweight and obese adults and could experience an earlier onset of chronic diseases such as type 2 diabetes and CVD (3). Given the current obesity rates in U.S. children and adolescents (4), identifying modifiable risk factors underpinning excessive weight and adiposity gain early in life is urgently needed.

Early complementary feeding represents the progressive introduction of solid foods between 5 and 12 months of age as infants no longer rely solely on breastmilk or formula. Growth trajectories and shifts in the composition of the gut microbiota during this critical period have the potential to program long-term body weight, body composition, and disease risk, with diet exhibiting significant influence. Among the macronutrients, protein has gained great interest over the years. Multiple observational and randomized controlled trials (5–7) have evaluated protein content in liquid diet (i.e., infant formula) and most studies observed a lower-protein content in infant formula led to less weight gain and lower weight-for-length Z scores (WLZ). These findings at least partially contributed to the recommendations of reducing protein intake during complementary feeding (8). However, a significant knowledge gap exists regarding the effects of different protein-rich foods on growth during early complementary feeding.

Research of protein-rich complementary foods on growth and risk of overweight is quite limited. Several observational studies focused on the long-term effect of early protein intake, but results were inconsistent. One study in Iceland found that the intake of animal protein at 12 months (meat and dairy combined), but not plant-based protein, was associated with higher BMI at age 6. Importantly, dairy protein was also associated with higher IGF-1 (9), which is associated with a higher risk of obesity early in life (10). A larger study in the Netherlands had similar findings, and the association between animal protein and BMI did not differ between meat and dairy (11). However, another study from Germany found that dairy intake at 12 months was associated with BMI at age 7, while meat or plant was not (12). A 2019 Cochrane review on animal-source foods for growth and development in infants and young children rated the quality of the current evidence as very low overall and no firm conclusions can be drawn (13). Likewise, a 2019 systematic review by the B-24 committee concluded that there is insufficient evidence of an association between protein intake and incidence of overweight or obesity early in life (14).

Another health indicator that might link diet and growth is the gut microbiota. The role of gut microbiota in human health, including obesity risks, has been examined primarily in adults and animal models (15, 16). Emerging research suggests that early-in-life colonization plays a critical role in the establishment and maturation of gut microbiota, and disruption of the optimal microbial succession may result in long-term health impairments (17). Although gut microbiota are greatly influenced by diet, very few studies have addressed the effects of complementary foods on infant gut microbiota. Several observational studies (18) showed significant shifts of both diversity (19) and community structure of the gut microbiota during weaning and dependent on types of complementary foods consumed (20). One animal study (21) compared meat-, dairy- and plant-protein extracts and found *Ruminococcaceae* was one of the characteristic bacteria in rats fed with meat proteins. A previous study from our group in 6–9 month-old infants showed that compared with a low-protein, cereal-based complementary diet (9% energy from protein), a high-protein, meat-based diet (17% energy from protein) increased the abundance of short-chain fatty acids (SCFA)-producing *Lachnospiraceae* (22). This project will directly assess the development of gut microbiota during the transition from liquid diets (breastmilk or formula) to complementary feeding, and in response to different protein-rich foods.

Emerging research has shown that gut microbiota can directly regulate growth. One animal study (23) found that gut microbiota drives growth during the juvenile period. A landmark cohort study (24) identified bacterial species whose proportional representation defined a healthy and mature gut microbiota during the first year of life in Malawian infants. Specifically, deviation from the normal gut microbiota, such as low diversity and absence of certain species, resulted in “immature” gut microbiota and growth impairment (24). Furthermore, transplanting gut microbiota from stunted infants to germ-free mice also transmitted impaired growth phenotypes, and adding back the two major growth-discriminatory species (*Ruminococcus gnavus, Clostridium symbiosum*) ameliorated growth impairment in mice (24). *Ruminococcus* and other short-chain fatty acids (SCFA) producing strains may increase the gut SCFA content, which, in animal models, could directly promote bone growth (25). Two recent cohort studies found that disrupted maturation of the gut microbiota, as indicated by low diversity, is associated with growth failure in preterm infants (26) and slower growth in weight in Malawian infants (27). However, a recent cohort study (28) found that the abundances of SCFA producing families *Lachnospiraceae* and *Ruminococcaceae* at 4 months were associated with a higher risk of overweight at 12 months. Current findings, although limited and primarily in animal and cohort studies, suggest that gut microbiota could impact growth and risk of overweight. The project presented here will directly assess the relation of gut microbiota and infant growth longitudinally and its potential mediating effect.

### Objectives and Hypotheses

#### Goal

The goal of this randomized controlled feeding trial is to establish how infant diet with different protein-rich foods affects growth trajectories and gut microbiota development during the early complementary feeding phase.

#### Aims

The objective of this study is to determine the impact of different types of common protein-rich foods on infant growth (Aim 1) and gut microbiota (Aim 2). We also seek to identify the role of the gut microbiota in infant growth (weight, length, head circumference, adiposity), specifically, whether the gut microbiota is a mediator linking protein-rich foods and infant growth (Aim 3).

#### Hypotheses

Primary: Growth trajectories (weight, length and body composition) and gut microbiota (diversity and composition) during early complementary feeding will differ by types of protein-rich foods consumed. Specifically, meat- and plant-based diets will have a lower weight-for-length Z score and adiposity, more age-appropriate and diverse gut microbiota than dairy and the reference groups. Secondary: A more mature, diverse microbiota will be positively associated with infant growth and that diet-induced infant growth trajectories will be mediated by the gut microbiota.

## Methods

### Study Design

**Figure 1:**
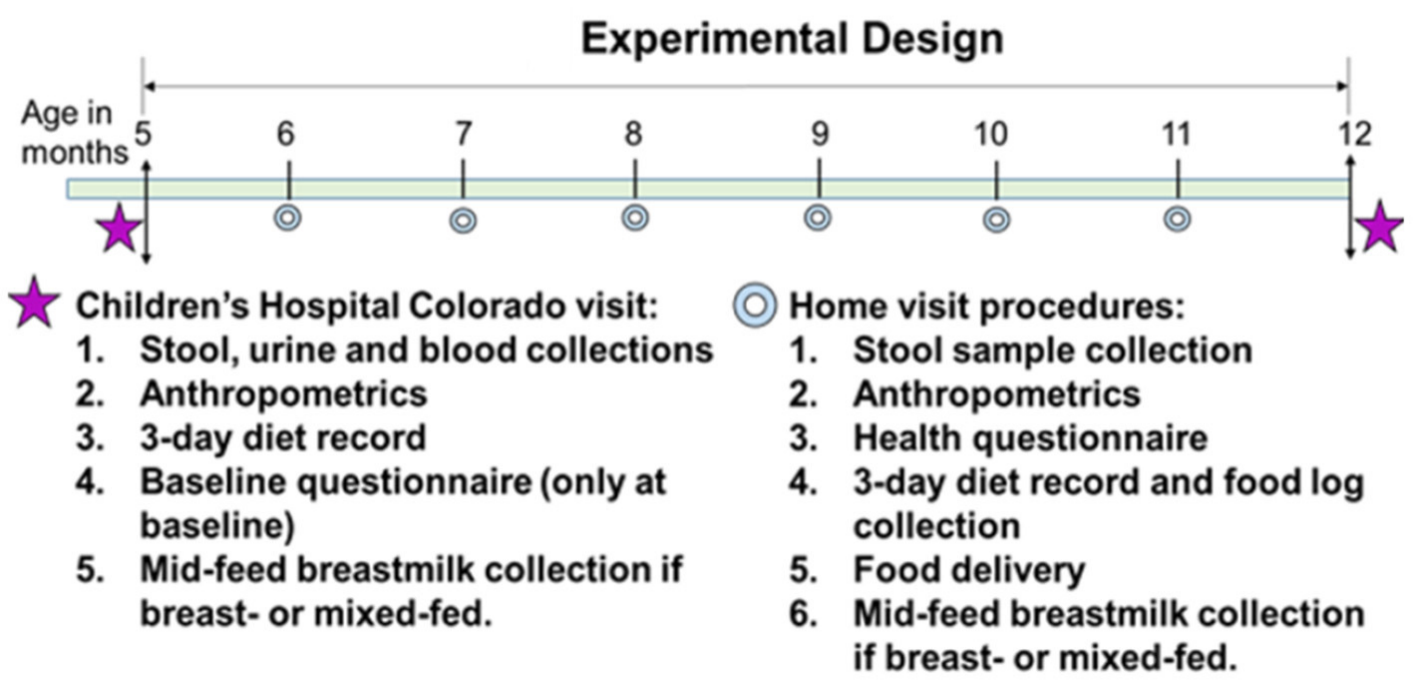
Experimental design.

This is a randomized controlled feeding trial with four groups. After screening, eligible participants and their caregiver(s) will visit the Clinical & Translational Research Center (CTRC) at Children's Hospital Colorado (CHCO) to complete the baseline visit (Figure 1) at 5 months of age. After obtaining informed consent, participants will be assigned and randomized to one of the four study diets: (1) a meat-protein-predominant diet group (Meat); (2) a dairy-protein-predominant diet group (Dairy); (3) a plant-protein-predominant diet group (Plant); or (4) a reference group without intervention (Reference).

The baseline visit includes consenting to study participation, a questionnaire of infant feeding and family health history, family demographics, parental weight and height, gestational weight gain, parity, maternal smoking, and other variables that could affect outcomes. Blood, urine and stool samples are collected. Urine samples are used for body composition and total energy expenditure assessments using the doubly labeled water method with procedures designed for infants. A fasting breastmilk sample is also collected at baseline. When the intervention ends at 12 months of age, participants come to Children's Hospital Colorado CTRC to complete the end of the intervention visit. During the intervention (5–12 months), monthly home visits are conducted to (1) deliver study foods to Meat, Dairy and Plant and grocery vouchers to Reference, and infant formula if formula-fed or mixed feeding; (2) complete a health and dietary questionnaire; (3) obtain length and weight measurements; (4) collect a stool sample; (5) collect 3-day diet record and the food tracking log; (6) collect a breastmilk sample if breastfeeding.

### Timeline and Milestones (in Months, Commencing March 1, 2021)

0–5 Study preparations: Manuals of operation, IRB approval, Children's Hospital Colorado Research Institute Approval and Clinicaltrials.gov registration.
6–48 Enrollment of 260–300 eligible participants and complete all baseline visits.
21 Completion of 50% participants in each group complete.
54 All clinical procedures complete.
60 Laboratory analyses of blood, urine and stool analyses, data analyses complete.

### Participants

Participants are being recruited in the metro Denver area from households with 3–4 month-old infants via direct mailing by Colorado Department of Public Health & Environment (CDPHE) which has access to the birth registry. Up to 300 infants will be recruited (75/group) to have at least 240 completers (60/group), based on a conservative estimate of 20% attrition.

#### Inclusion Criteria

Full term (gestational age equal or over 37 weeks); generally healthy without conditions that would affect protein metabolism or growth; no previous complementary food exposure; no prior exposure of antibiotics during delivery or after birth; able to consume study foods; No known food allergies.

#### Exclusion Criteria

Pre-term or small-for-gestational age infants; Infants having conditions that would affect normal growth; Infants having had complementary foods prior to the start of the study; not willing to feed the complementary foods provided; antibiotics exposure during delivery or from birth to 5 months of life; multiple births.

### Ethical Approval

Ethical approval for this clinical trial was obtained through the Colorado Multiple Institutional Review Board (COMIRB) (COMIRB 20-2232). COMIRB conducts the initial and annual reviews of the trial, monitoring enrollment and retention, protocol deviations, and any reported adverse effects as a result of the study. All participants are provided with a copy of the signed informed consent. This protocol is registered at ClinicalTrials.gov NCT05012930.

### Study Procedures

#### Enrollment

Participant enrollment begins before 5 months of age. Interested participants are screened by phone or email; if eligible, an in-person baseline visit is scheduled and informed consent is obtained at the baseline visit. Enrollment of eligible infants take place over the first 4 years of the study. As part of the informed consent process, participants are notified that they may withdraw at any time and of the actions that may deem the infant ineligible for the study. At time of enrollment, each participant is assigned a unique study-ID (MINT-001 through MINT-300) for subsequent data collection.

#### Randomization

Upon enrollment, participants are randomized into one of four groups, differentiated by source of protein-rich foods they consume (meat, dairy, plant or control group). A stratified random sampling design is used where the strata are defined by mode of feeding (breastfed or formula-fed), sex (male or female) and mode of delivery (cesarean or vaginal delivery). The four feeding groups are randomized within each strata in groups of four so each diet (Meat, Dairy, Plant, Reference) occurs exactly once per four enrollments. Given our rolling recruitment strategy over 4 years, this ensures that the initial randomization of group within each strata is close to balanced and that there is little to no relationship with the order of recruitment. A stratified randomization design controls for possible confounding variables and results in an increase in power.

#### Baseline Data to Be Collected Includes

##### Demographic Information

Study participants are given a questionnaire at the baseline visit to report both the infant and parental ethnicity, race, gender, high-risk behaviors such as smoking tobacco or other recreational drugs, education level, and gross family income.

##### Infant and Paternal Health History

Another questionnaire is given to the caregiver to discuss birth details such as mode of delivery and infant weight and length. Caregivers are asked to disclose the family health history of the following: obesity, cardiovascular disease, type 2 diabetes mellitus, and allergies in immediate family members. Infant feeding habits for the previous month are also assessed in addition to any illness, medication, supplements, or antibiotic use. This infant health questionnaire is to be repeated at each subsequent monthly home visit.

##### Anthropometric Measurements

Pediatric nurses at the CTRC, who are blinded to the treatment groups, obtain three measurements of weight in kilograms, three measurements of length in centimeters, and three measurements of head circumference in centimeters. Study coordinators calculate the average measurement as the baseline anthropometrics for the participant. Parental self-reported height and weight measurements are also to be obtained at this visit.

##### Dietary Intake

A weighed 3-day diet record is collected from caregivers before the intervention begins. A kitchen scale and calibration weight, plus the diet record with instructions, are provided to caregivers. For breastfed infants who do not use bottles, testing weighing using Seca^TM^ 757 infant scale is conducted before and after each feed during the 3 days when the 3-day diet record is filled out. Registered dietitians from the research team train parents/caregivers to fill out the diet record.

##### Biospecimen Samples

At the baseline visit, pediatric nurses at the CTRC draw blood from all able participants via venipuncture (3–5 ml). Samples are centrifuged, and serum is stored at −80°C until analyzed. Individual aliquots will be used to measure ICF-1, ICFBP3, insulin, blood lipids (triglycerides, HDL, LDL and total cholesterol), alpha 1-acid glycoprotein (AGP), high sensitivity C-reactive protein (CRP), and quantitative amino acids. After the baseline visit at the Children's Hospital Colorado CTRC, urine samples (one pre-dose and seven post-dose) are collected by the caregiver to assess total energy expenditure and two-compartment body composition using the doubly labeled water technique (29). Urine samples are collected by placing cotton balls in the diaper and after soaked with urine, cotton balls will be placed in the barrel of a 20 ml sterile syringe and urine will be expressed into a sterile plastic tube. A stool sample is also collected from participants' homes for gut microbiota analysis. Stool samples are collected by placing a diaper liner in the infant's diaper, which allows urine to pass and stool to stay. Caregivers are advised to collect the soiled diaper liner and keep it in the home freezer before the study coordinator retrieves it. Lastly, a fasting breastmilk sample is collected from caregivers who are breastfeeding. Macronutrient and energy profiles of breastmilk are assessed via mid-infrared spectroscopy (Human Milk Analyzer, Miris, Uppsala, Sweden). Detailed instructions on how to collect urine, stool and breastmilk samples, and sample collection kits are provided to caregivers.

### Dietary Intervention

Participants in the three intervention groups are asked to avoid protein-rich foods from other assigned groups (30) and consume protein only from their assigned group. All complementary foods, including infant cereal, low-protein fruits and vegetable purees, and designated protein-rich foods are provided to the three intervention groups. For formula-fed infants, the same brand cow-milk based infant formula is provided. The most recent NHANES report showed that the median protein intake of US infants 6–11 months (31) is 10% total energy or 2.5 g/kg/d. This quantity is used as the targeted total protein intake. Caregivers of infants in the reference/control group are given compensation to purchase complementary foods. Based on weight recorded at the visit and the reported formula/breastmilk intake, a recommended amount of meat- dairy- or plant-based food will be provided to caregivers to approximate a total protein intake of 2.5 g/kg/d. This estimation is updated every month. A sample diet plan is in Table 1, based on a formula-fed infant with a 60th percentile weight-for-age at 9 months.

**Table 1:** Examples of diet plans for the three intervention groups^a^.

	**Food item**	**Protein**	**Calorie**
Total	Total calorie needs		~650 kcal/d
	Total protein (2.5 g/kg/d)	21 g/d	84 kcal/d
Formula (not restricted)	Formula 18 ounces	9 g/d	400 kcal/d
Dairy group^b^	1 Yogurt (Yobaby^®^)	4 g/d	80 kcal/d
	Cheese (shredded) (Horizon^®^)	6.5 g/d	80 kcal/d
	Others (e.g., 1 Beech Nut^®^ zucchini and banana blend; or one servicing of fortified rice cereal)	1 g/d	
Meat group^b, c^	1.5 jar of ham and gravy (Gerber^®^)	10 g/d	80 kcal/d
	Others (e.g., 1 Beech Nut^®^ zucchini and banana blend; or one servicing of fortified rice cereal)	1 g/d	
Plant group^d^	1.5 vegetable pouch (e.g., Ella's kitchen^®^ four bean feast)	5 g/d	90 kcal/d
	1.5 vegetable pouch (e.g., Earth's best^®^ spinach lentil)	5 g/d	50 kcal/d
	Others (e.g., 1 Beech Nut^®^ zucchini and banana blend; or one servicing of fortified rice cereal)	1 g/d	
Reference group	No restriction (observational group and will follow standard of care)		

a *These estimates are based on an exclusively formula-fed 9-month-old female with a 8.5 kg body weight which is ~60th percentile weight-for-age*.

b *Both Dairy and Meat groups are advised to avoid plant foods of relatively high protein contents (a list will be provided). Cheese will be shredded before providing to the infant*.

c *The Meat group is allowed to have fish. However, commercial fish-based infant foods are very rare and if parents choose to feed home-made fish to the participants, they will record the time, type and amount*.

d *Both dairy- and plant-based complementary foods have low iron content. Participants in all intervention groups are advised to consume one serving of iron-fortified cereal per day to meet their iron needs*.

### Follow-Up Procedures: Home Visits

Monthly home visits at ages 6, 7, 8, 9, 10, and 11 months of age (±7 days) are conducted for each participant by one or two study coordinators. At each home visit, coordinators collect the following: stool sample (pre-collected by parents within 48 h before the visit), anthropometric measures (weight, length, head circumference), health questionnaire, 3-day diet record, food intake log, and breastmilk collection if the participant consumes breastmilk. The 9-month diet record is a weighed 3-day diet record. Caregivers also conduct test weighing again at 9 months if the participants consume breastmilk. Study coordinators also deliver study food (intervention groups) or food voucher (reference group) at the visit. Growth parameters are plotted on the infant's growth chart and Z-scores are calculated.

### Follow-Up Procedures: 12-Month and 24-Month Visits

At 12 months of age (±7 days), the study participant returns to the Children's Hospital CTRC to complete their end of intervention visit. The visit includes the same procedures and sample collections as the baseline visit. The dietary intervention ends at 12 months. At the 12-month visit, caregivers are asked whether they want to consent to a 24-month follow-up visit. If so, caregivers sign another consent for the 24-month follow-up. When the participant turns 24 months, 1 year after the intervention ends, they come back to the Children's Hospital CTRC and repeat the procedures at baseline and 12 months, except for body composition and total energy expenditure assessment.

### Primary Outcomes

1) *Infant growth*

Pediatric nurses at the Children's Hospital Colorado CTRC (at baseline, 12 and 24 months) and study coordinators (at monthly home visits) obtain a series of infant anthropometric measurements. These measurements including weight (Seca^TM^ 337 infant scale), length (obtained in recumbent position using an infant stadiometer), and head circumference-for-age (Seca infant head circumference measuring tape). Growth Z scores are calculated using length and weight based on WHO/CDC standards (32).

2) *Body composition and total energy expenditure at 5 and 12 months of age*

At baseline and 12 months, a pre-dose urine sample is collected to document basal enrichment of ^2^H and ^18^O, then 0.1 g/kg body weight 99% enriched ^2^H_2_O and 0.3 g/kg 10% enriched H${}_{2}^{18}$O are orally administered. Caregivers are asked to collect one daily sample for 7 days post-dosing. Urine samples are analyzed for ^18^O and ^2^H enrichment by Off-Axis Integrated Cavity Output Spectroscopy (OA-ICOS, Los Gatos Research Inc., Mountain View CA) (33). Isotope dilution space will be calculated via the standard equation of pre-dose and zero-time intercept enrichment via back-extrapolation, which is validated in infants (34). Total body water will be calculated from dilution space × 1.04 to account for water exchange in non-aqueous tissues. Finally, total fat-free mass will be obtained from total body water ÷ age/sex-specific hydration factor (i.e., 79% for 6-month-old infants). CO_2_ production, obtained from the fractional turnover rates of ^2^H and ^18^O (35), is converted to total energy expenditure via Weir equation (36).

3) *Gut microbiota*

Caregivers will obtain a fresh stool sample each month of participation (5–12 months of age) and one at 24 months. 16S rRNA gene amplicon sequencing (37–39) will be performed on all samples to profile fecal bacterial communities. Following sequence QA/QC (37–39), 16S rRNA gene sequences are classified using the SINA/Silva classifier.

Shotgun metagenomic sequencing (40) will be performed on baseline and 12-month samples to determine the functional genomic capacity of fecal microbiota and broaden taxonomic profiles to include archaea, bacteriophage, DNA viruses, and microbial eukaryotes. Following sequence QA/QC, ***[1–4]*** sequences will be co-assembled with MEGAHIT ***[5]***, open reading frames predicted (Prodigal ***[6]***), and annotated using DIAMOND ***[7]*** to query the NCBI non-redundant database. Bacterial species and strains are inferred using MIDAS ***[8]***.

All sequences and associated metadata will be deposited in the NCBI sequence read archive, following the MIMARKS standard.

### Secondary Outcomes

1) *Dietary intake*

Three-day diet records are analyzed by the CTRC Nutrition Core using the NDSR software (Minneapolis, MN) by a trained dietitian. The Food and Nutrient Database in NDSR contains over 18,000 foods, including brand-name infant foods used in the proposed study. Outputs include total energy, carbohydrate (fiber, fructose, glucose, sucrose, starch, etc.), fat (cholesterol, different kinds of fatty acids), protein (meat, dairy, plant, breakdown of amino acids), vitamins and minerals. Macronutrient and energy profiles of breastmilk samples are assessed via mid-infrared spectroscopy (Human Milk Analyzer, Miris, Uppsala, Sweden).

2) *Blood biomarkers*

IGF-1, IGFBP3, insulin, blood lipids, alpha 1-acid glycoprotein (AGP), high sensitivity C reactive protein (hsCRP) and quantitative amino acids will be measured at the University of Colorado Anschutz Medical Campus CTRC Core lab (30).

### Power Calculation and Sample Size Justification

We hypothesize that both the gut microbiota and infant growth trajectories are dependent on types of protein-rich foods consumed. We will recruit up to 75 infants/arm and use a conservative 20% attrition which results in 60/group (total completers *n* = 240). Sample size and power justification are from the longitudinal models with feeding group as the predictor and either gut microbiota (i.e., diversity and *Ruminococcus* abundance) and growth (i.e., linear growth parameter LAZ, overweight parameter WLZ and growth velocity using the WHO 0–24 months standards) as the outcomes. We completed power simulations (1,000 replicates) using a linear mixed-effects model (LME) with random effects by subject. For the simulations, we used parameter estimates (e.g., fixed and random effects) from our randomized pilot study on the gut microbiota and infant growth trajectories after receiving meat- or dairy-based complementary diet (30). We simulated a variety of scenarios assuming that Plant had microbiota and growth outcomes that were either worse than Dairy, between Dairy and Meat, or the same as Meat. *To be conservative, we estimated power after increasing the within-group variability by 50%*. The power is above 80% in all simulated scenarios (80–90%) where the effect size was simulated to be at least 50% of that observed in our pilot study (30). The power of most simulation scenarios was below 80% when the effect size was 25% of that observed in the pilot study (30).

### Statistical Approach

Aim 1: Using ANOVA for continuous variables and chi-squared test for categorical variables, we will assess whether matching and randomness held by testing whether potential confounders (e.g., feeding mode, maternal height, maternal BMI, education, sex, race/ethnicity, and smoking during pregnancy) differ between groups. Variables with a *p* < 0.05 and those that have a strong association with the outcome of interest are included in future models. To model the relationship between diet, time, and diet by time interaction and LAZ and WLZ, we use LME model with random effects by subject. This structure explicitly models multiple time points per infant and can adjust for changes of cofounders during the intervention (e.g., a participant changed from breastfeeding to formula feeding at 9 months). Given a significant diet by time interaction, we will test pairwise differences in growth trajectories using Tukey's multiple comparisons.

Aim 2: We model the effects of diet on the gut microbiota at the end of the intervention and longitudinally, incorporating likely covariates and potential cofounders. 16S amplicon sequencing and metagenomic sequencing will each generate tables of annotated sequence counts. Both datasets will be used to model the diet's impact on microbiota taxonomic composition and functional capacity (i.e., genes and pathways identified through shotgun metagenomics) over the course of the intervention. To evaluate differences in overall taxonomic/functional community composition (i.e., beta-diversity), we use the Microbiota Regression based Kernel Association Test (MiRKAT). MiRKAT models the microbiota using phylogenetic kernels to account for differences in microbial profiles (16S or metagenomics count data) (41). As different measures of dissimilarity are optimal under different conditions, optimal-MiRKAT allows multiple dissimilarity metrics to be tested simultaneously. To model microbial taxonomic and functional profiles over time, we use two methods recently proposed (42). First, we use a bi-exponential distribution to summarize microbiota diversity curves over time. To make inferences, we will incorporate the distribution into a hierarchical model with a random intercept to account for multiple measurements for each person. This approach will model changes in individual taxa, both the rare and common, over time. Additionally, we will evaluate longitudinal changes in common alpha-diversity metrics (e.g., richness, evenness, Shannon diversity, and effective species [e.g., Hill's number (42)]). Finally, we use the control group to build a gut microbiota maturation model via a random forest approach as previously described (24) and compare the intervention groups to this model.

**Figure 2:**
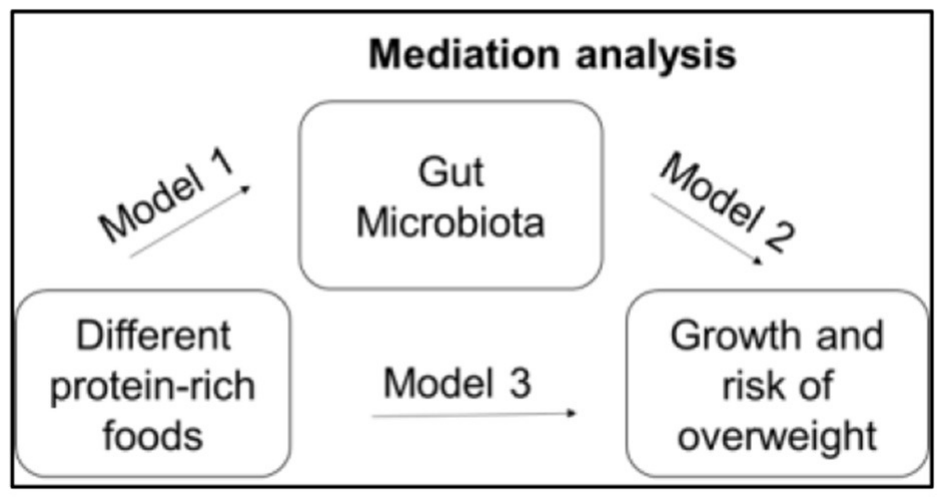
Mediation analysis.

Aim 3: We use LME on growth Z scores and the microbiota over time with random effects by subject, and include group as a covariate. We also complete secondary analyses stratified by group to identify relationships between Z scores and microbiota that differ by group. We will use microbiota diversity measures and relative abundances of individual taxa and genes/pathways to model diversity and composition of the microbiota. We also include necessary confounders, e.g., change from breastfeeding to formula. To evaluate whether taxonomic and/or functional features of the microbiota mediate the relationship between diet and growth, we use a method and R package called MedTest (43). As in classic mediation analysis, MedTest evaluates three models (44). In reference to Figure 2, the first model estimates the direct path between protein-rich foods and the gut microbiota and implements as part of Aim 1 using MiRKAT. The second model estimates the path between protein-rich foods and infant growth and will be implemented using a standard linear model in R. The third model estimates the path between protein-rich foods and growth controlling for the path between gut microbiota and growth and will be implemented using MiRKAT. Using these three models, MedTest tests for significances of the mediation effect of the gut microbiota. Since it is unknown which microbiota features may mediate the relationship, MedTest implements multiple microbiota distances: unweighted (45), weighted (46), and generalized UniFrac (47) as well as Jaccard and Bray-Curtis distances. To ensure a well-controlled type-I error rate, MedTest uses a modified permutation procedure to arrive at an omnibus test over all distances.

## Discussion

### Innovation and Significance

We focus on a critical developmental phase that has not been well-studied and propose a novel concept that during early complementary feeding, types of common protein-rich foods will differentially impact infant growth and risk of overweight and that gut microbiota will mediate this impact. To our knowledge, this is the first RCT that directly compared meat, dairy and plant consumptions on infant growth and gut microbiota. We also propose to use non-invasive body composition assessment with doubly labeled water, which also provides a reliable estimation of energy expenditure, a crucial component to assess the risk of overweight. Furthermore, we propose a plausible mechanism that protein-rich foods from different sources could impact infant growth trajectories by modulating the gut microbiota. Overall, this project is innovative because it is a substantively different concept and approach. It will greatly support implementing effective dietary interventions to prevent undesirable infant growth patterns and long-term negative health impact.

### Compliance Monitoring

Although we will use controlled feeding, we are aware that compliance with the dietary regime is a major challenge. Building on extensive experience, we will utilize the following strategies to optimize and monitor compliance: (1) A weekly multiple-choice log to record food intake based on the monthly recommendations (Table 1) as described in our pilot study (30). (2) Caregivers will be asked to return unconsumed foods/formula at the monthly home visit. (3) Weighed 3-day diet record will be collected at 5, 9, and 12 months; (4) Blood urea nitrogen (BUN), measured at 5 and 12 months is a crude marker of total protein intake, and values of BUN will reflect the total amount of protein consumed for comparisons between groups and over time. We also understand the study procedures may be burdensome to some caregivers. Our team has successfully conducted a number of infant trials with similar or more extensive procedures and low drop-out rate (30, 39, 48, 49). Our experienced team members are dedicated to fully support caregivers and will be available by phone/email and in person, and will make extra home visits if needed.

### Risks

The study protocol is expected to have minimal risk because there are no invasive procedures except the three blood draws at 5, 12, and 24 months of age, which are necessary to answer the research question. The 12-month blood draw will include analyses (Pb and Hb) routinely obtained for well-child surveillance; results will be provided to the primary care provider to avoid a second clinical blood draw. There is an unlikely risk of food allergies. During screening, the caregiver is asked if the infant has any food restrictions or allergies, including milk/dairy protein allergies (i.e., on a special formula). If the answer is yes, the infant is not eligible to participate. If the participant has no food restrictions or allergies, he/she is unlikely to be allergic to the complementary foods provided because most of the study foods are not considered highly allergenic foods. At the baseline visit, the study coordinator explains the potential risk of allergy development when starting complementary feeding and caregivers are given a list of common signs of food allergies. Food allergy is closely monitored by monthly health questionnaires. If repeated concerns are expressed, the participant will be removed from the study and advised to consult the primary care physician.

## Author Contributions

MT conceived of the trial. NK, EM, DF, and AH supported the design and development of the trial. KM, LB, and KD contributed to finalizing the manuscript. All authors contributed to the article and approved the submitted version.

## Funding

This work was supported by NIH R01DK126710.

## Conflict of Interest

The authors declare that the research was conducted in the absence of any commercial or financial relationships that could be construed as a potential conflict of interest.

## Publisher's Note

All claims expressed in this article are solely those of the authors and do not necessarily represent those of their affiliated organizations, or those of the publisher, the editors and the reviewers. Any product that may be evaluated in this article, or claim that may be made by its manufacturer, is not guaranteed or endorsed by the publisher.
